# Duck Enteritis Virus Glycoprotein D and B DNA Vaccines Induce Immune Responses and Immunoprotection in Pekin Ducks

**DOI:** 10.1371/journal.pone.0095093

**Published:** 2014-04-15

**Authors:** Yan Zhao, Yongsheng Cao, Lihong Cui, Bo Ma, Xiaoyu Mu, Yanwei Li, Zhihui Zhang, Dan Li, Wei Wei, Mingchun Gao, Junwei Wang

**Affiliations:** 1 Group of Avian Respiratory infectious Diseases, State Key Laboratory of Veterinary Biotechnology, Harbin Veterinary Research Institute, the Chinese Academy of Agricultural Sciences, Harbin, China; 2 Northeast Agricultural University, Harbin, China; Thomas Jefferson University, United States of America

## Abstract

DNA vaccine is a promising strategy for protection against virus infection. However, little is known on the efficacy of vaccination with two plasmids for expressing the glycoprotein D (gD) and glycoprotein B (gB) of duck enteritis virus (DEV) in inducing immune response and immunoprotection against virulent virus infection in Pekin ducks. In this study, two eukaryotic expressing plasmids of pcDNA3.1-gB and pcDNA3.1-gD were constructed. Following transfection, the gB and gD expressions in DF1 cells were detected. Groups of ducks were vaccinated with pcDNA3.1-gB and/or pcDNA3.1-gD, and boosted with the same vaccine on day 14 post primary vaccination. We found that intramuscular vaccinations with pcDNA3.1-gB and/or pcDNA3.1-gD, but not control plasmid, stimulated a high frequency of CD4^+^ and CD8^+^ T cells in Pekin ducks, particularly with both plasmids. Similarly, vaccination with these plasmids, particularly with both plasmids, promoted higher levels of neutralization antibodies against DEV in Pekin ducks. More importantly, vaccination with both plasmids significantly reduced the virulent DEV-induced mortality in Pekin ducks. Our data indicated that vaccination with plasmids for expressing both gB and gD induced potent cellular and humoral immunity against DEV in Pekin ducks. Therefore, this vaccination strategy may be used for the prevention of DEV infection in Pekin ducks.

## Introduction

Duck viral enteritis (DVE) is an acute contagious disease in all types of birds, including ducks, geese, and swans [1]. DVE is caused by duck enteritis virus (DEV), which was first known as an acute hemorrhagic disease in domestic ducks in Holland in 1923 [2]. Outbreaks of DVE were reported in many countries, including in China in 1957 [3]. Studies indicate that DVE survivors may carry the virus up to four years. DEV infection can establish an asymptomatic carrier state in both farmed and wild waterfowl [4]. DEV is a member of the family Herpesviridae, according to the Ninth International Committee on the Taxonomy of Viruses (ICTV) [5], and DEV can cause severe clinical symptoms, such as inappetence, extreme thirst, droopiness, ataxia, ruffled feathers, nasal discharge, watery diarrhea and a drop in egg production, leading to high mortality in ducks and a huge economic loss in the industry. Therefore, the development of new vaccines for the prevention of DEV infection is crucial for control of DVE in birds.

The effectiveness of a vaccine is dependent on the immunogenicity of antigens vaccinated. Glycoprotein D (gD) is one of the several surface glycoproteins on the viral membrane and is an essential component for viral entry into host cells [6], [7]. Previous studies have shown that the gD is a major antigen for the design of vaccines [8], [9] and that vaccination with protein subunit of gD protects from herpetic diseases in animals [10], [11], [12]. Glycoprotein B (gB) is one of the major glycoproteins in the viral envelope and plasma membrane of virus-infected cells. The gB is essential for attachment and penetration of free virions [13], involving fusion between the virion envelope and the cellular plasma membrane [14], as well as direct transmission of infectivity from primary infected cells to neighboring non-infected cells [15], [16]. In many herpesviruses, the gB homolog has been reported to elicit neutralizing antibodies in mammals and birds [17], [18], [19], [20], [21], [22], [23], [24]. Accordingly, we hypothesized that vaccination with subunit of gB could protect animals from DEV infection. However, it is unknown whether simultaneous vaccination with both antigens could synergistically enhance immunity and protect from DEV-mediated diseases in Pekin ducks.

DNA vaccines are not only safe, easy to preparation and use, but effective and inexpensive. In this study, we generated two plasmid DNA vaccines for expressing gB and gD, respectively, and examined the efficacy of vaccination with monovalent or bivalent vaccines in inducing immune responses and protection against virulent virus-mediated disease in Pekin ducks. Our data indicated that vaccination with bivalent vaccines induced potent immunity and strong protection against DEV infection in Pekin ducks.

## Materials and Methods

### Ethics Statement

Animal experiments were approved by Animal Ethics Committee of Harbin Veterinary Research Institute of the Chinese Academy of Agricultural Sciences (CAAS) and performed in accordance with animal ethics guidelines and approved protocols. The Animal Ethics Committee approval number was SYXK (Hei) 2011022.

### Cells and virus

The commercial DEV vaccine, DEV C-KCE, used in China and virulent strain of DEV AV1221 were from the China Institute of Veterinary Drugs Control (Beijing, China). The DEV C-KCE strain was propagated in chicken embryo fibroblast (CEF) cells cultured in Dulbecco's Minimal Essential Medium (DMEM) containing 10% fetal bovine serum (FBS, Invitrogen, USA). Viral particles were harvested when the cytopathic effect reached 80%. The infected cells containing DEV virions were subjected to three cycles of freeze-thaw and centrifugation, and stored at –70°C until use. The virulent strain AV1221 was used for challenge.

### Plasmid construction, purification and expression in vitro

The plasmid pcDNA3.1-gB encoding the full-length sequence of DEV gB was constructed by our laboratory. To generate the plasmid pcDNA3.1-gD, a DNA fragment for the gD gene was amplified from the DEV C-KCE genome by PCR using the specific primers of sense: 5′ AAGCTTGCAGCTATGGGCAATCATG3′, and antisense: 5′ GAATTCTGTATTTTATGTCCACATAGGCGC3′ that were designed based on the gD gene (GenBank accession no. EU621392). The PCR products were digested with restriction enzyme *Eco*RI and *Hin*dIII (TaKaRa, Dalian, China) and cloned into the corresponding sites of pcDNA3.1 (+) vector (Invitrogen). The authenticity of the pcDNA3.1-gD and pcDNA3.1-gB were characterized by DNA sequencing, and all new data has been deposited in GenBank. Subsequently, pcDNA3.1-gD and pcDNA3.1-gB were transformed into *E. coli*, extracted, and purified by Macrogol8000 (Sigma, USA), followed by suspending in sterile phosphate-buffered saline (PBS) for immunization.

The expression of gB and gD was characterized by indirect immunofluorescent assay (IFA). In brief, DF1 cells were transfected with pcDNA3.1-gB or pcDNA3.1-gD using lipofectamine 2000 for 72 h. Subsequently, the cells were stained with mouse-anti-gB, rabbit-anti-gD polyclonal antibodies (generated by immunizing animals with the gB or gD purified from the gene-transfected cells in our laboratory), and then stained with FITC-goat-anti-mouse IgG, or FITC-goat-rabbit IgG (ZhongShanJinQiao, Beijing, China), respectively. The expression of gB or gD was observed under a fluorescent microscope.

### Immunizations and challenge

Fifty DEV-seronegative Pekin ducks at 10 weeks of age were randomly divided into five groups. The ducks were injected intramuscularly with 1 mg/kg body weight of 0.5% neocaine in the femoribus internus. Twenty minutes later, the ducks were vaccinated at the same location with 200 µg pcDNA3.1, pcDNA3.1-gD, pcDNA3.1-gB, combination of pcDNA3.1-gD and pcDNA3.1-gB in 200 µl of PBS or PBS alone (n = 10 per group), respectively. The Pekin ducks were boosted with the same dose of plasmid two weeks after the primary vaccination. Blood samples of individual Pekin ducks were obtained on days 7, 14, 21, and 28 post primary immunization for analysis of the levels of serum IgG antibodies against DEV, the titers of anti-DEV neutralizing antibodies, the frequency of CD4^+^ T and CD8^+^ T lymphocytes in peripheral blood cells. On day 28 post primary immunization, individual Pekin ducks were challenged intramuscularly with 10^4^ minimum lethal doses (MLD) of DEV AV1221 (MLD  = 10^−6.3^/ml). The clinical symptoms of individual Pekin ducks and the percentages of morbidity and mortality in individual groups were monitored and recorded up to 14 days after challenge. Autopsy was done soon after death.

### Enzyme-linked immunosorbent assay (ELISA)

The concentrations of serum IgG antibodies against DEV were tested by ELISA. Briefly, individual wells were coated with 33 ng/well of the purified DEV C-KCE and blocked by 1% gelatin and 0.05% polyvinyl alcohol. The coated DEV antigens were probed in triplicate with 1∶400 diluted serum samples at 37°C for 1 h. After washing, the bound antibodies were detected with 1∶400 diluted horseradish peroxidase (HRP)-conjugated goat anti-duck IgG (KPL, Maryland, USA) and visualized using 100 µl/well of 3,3′,5,5′- tetramethylbenzidine (TMB) chromogenic fluid, followed by reading absorbance at 450 nm and 630 nm using an microplate reader (SpectraMax plus 384, Molecular devices, USA). The sera from the PBS-injected control Pekin ducks and sera from a group of Pekin ducks injected with duck hepatitis virus were used as negative controls. The cut-off value for positive antibody response was determined by the mean value of negative sera plus two standard deviations.

### Microneutralization assay

The titers of neutralization antibodies against the virus in individual sera were determined before challenge [25]. Briefly, individual heat inactivated sera were subjected to serial dilutions (1∶2–1∶256) and mixed with 100 50% tissue culture infective doses (TCID_50_) of DEV at 37°C for 1 h, and the mixtures were incubated in quadruplicate with a monolayer of chicken embryo fibroblast cells (CEF) on 96-well tissue culture plates at 37°C for 1 h, followed by incubating with 200 µl DMEM for 3–4 days. The cells cultured with medium alone, with medium containing positive or negative serum alone, or with 50 µl of 100 TCID_50_ and 50 µl of 0.1 TCID_50_ virus suspensions were used as negative or positive controls. The virus-mediated cytopathic effects were determined when the positive control cells exhibited greater than 75% of cells with cytopathic effect. The titers of virus neutralization antibodies in individual sera were determined in a blinded manner.

### Detection of CD4+ and CD8+ T lymphocytes in peripheral blood

Blood samples were collected weekly after the primary immunization, and lymphocytes were isolated using lymphocyte separation medium (Sigma, USA), according to the manufacturers’ instruction. Subsequently, the isolated cells (1×10^6^/tube) were stained with mouse anti-duck CD4 or CD8 mAb (provided by Professor Bernd Kaspers, Munich University, Germany). After washing, the cells were further stained with 1∶200 diluted FITC-goat-anti-mouse IgG (ZhongShanJinQiao, Beijing, China) and characterized by flow cytometry analysis.

### Statistical analysis

Data are expressed as mean ± SD. The difference among groups was analyzed by two-way multivariate analysis of variance (MANOVA) and Tukey's multiple comparison tests using SPSS software. The death rates among the groups were analyzed by the Fisher exact test (FREQ process) and the survival periods among the groups of animals were analyzed by log-rank test (life-table process) using SARS8.0. A p value of less than 0.05 was considered statistically significant.

## Results

### Construction of plasmids for expressing gD and gB

**Figure 1:**
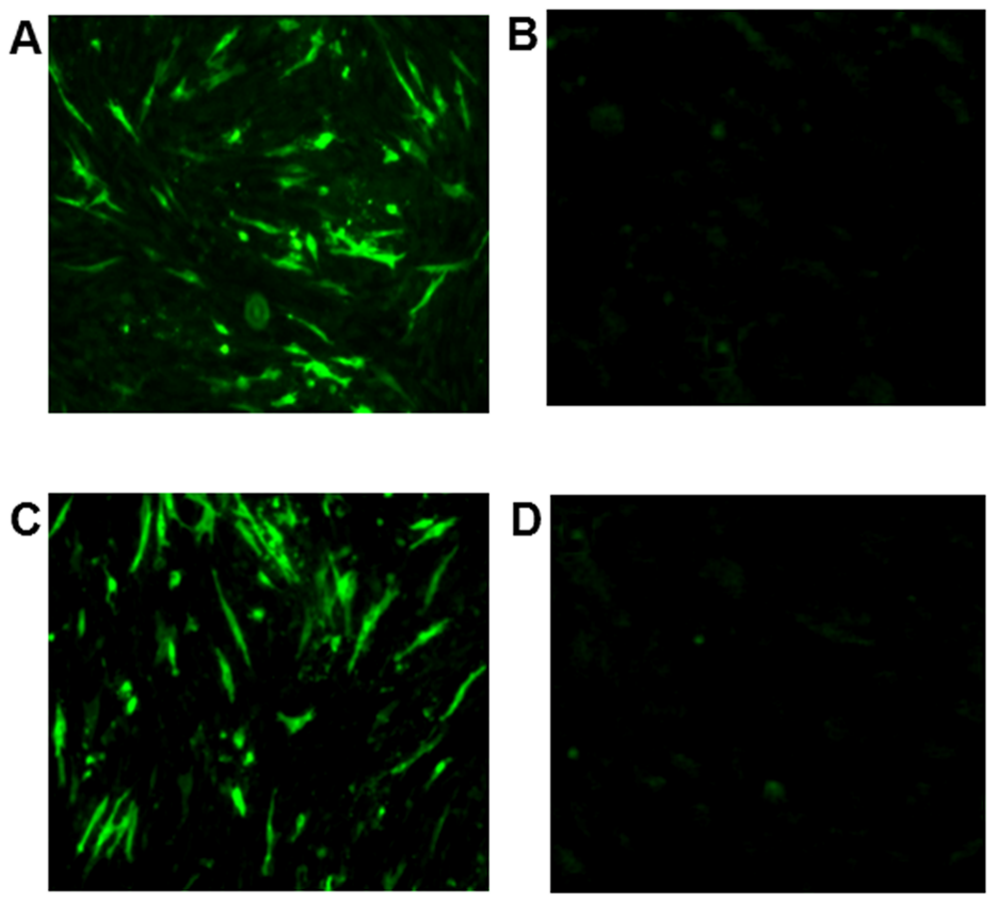
Characterization of gB and gD expression in DF1 cells. DF1 cells were transfected with plasmid of pcDNA3.1-gB or pcDNA3.1-gD for 72 h, and the expression of gB and gD was characterized by immunofluorescent assays using specific antibodies. Data shown are representative images of each group of cells from two separate experiments. A, B: DF1 cells transfected with pcDNA3.1-gB or pcDNA3.1(+) and stained with mouse-anti-gB and FITC-goat-mouse IgG; C, D: DF1 cells transfected with pcDNA3.1-gD or pcDNA3.1(+) and stained with rabbit-anti-gD IgG and FITC-goat-rabbit.

To generate plasmids for expressing gB and gD, the sequences of DEV gB and gD were amplified by PCR and directly cloned into pcDNA3.1 to construct pcDNA3.1-gB and pcDNA3.1-gD, respectively, followed by DNA sequencing. The obtained sequences were deposited in GenBank (Accession Nos. JN790940?JN790941). After transfection into DF1 cells, the expressed gB and gD were characterized by immunofluorescent assays. As shown in Figure 1, both gB and gD were predominantly expressed in the cytoplasma of DF1 cells. The successful generation of these two plasmids provides useful reagents for testing the immunogenicity of expressed proteins *in vivo*.

### Vaccination with both plasmids of pcDNA3.1-gB and pcDNA3.1-gD induces strong cellular immunity in Pekin ducks

**Figure 2:**
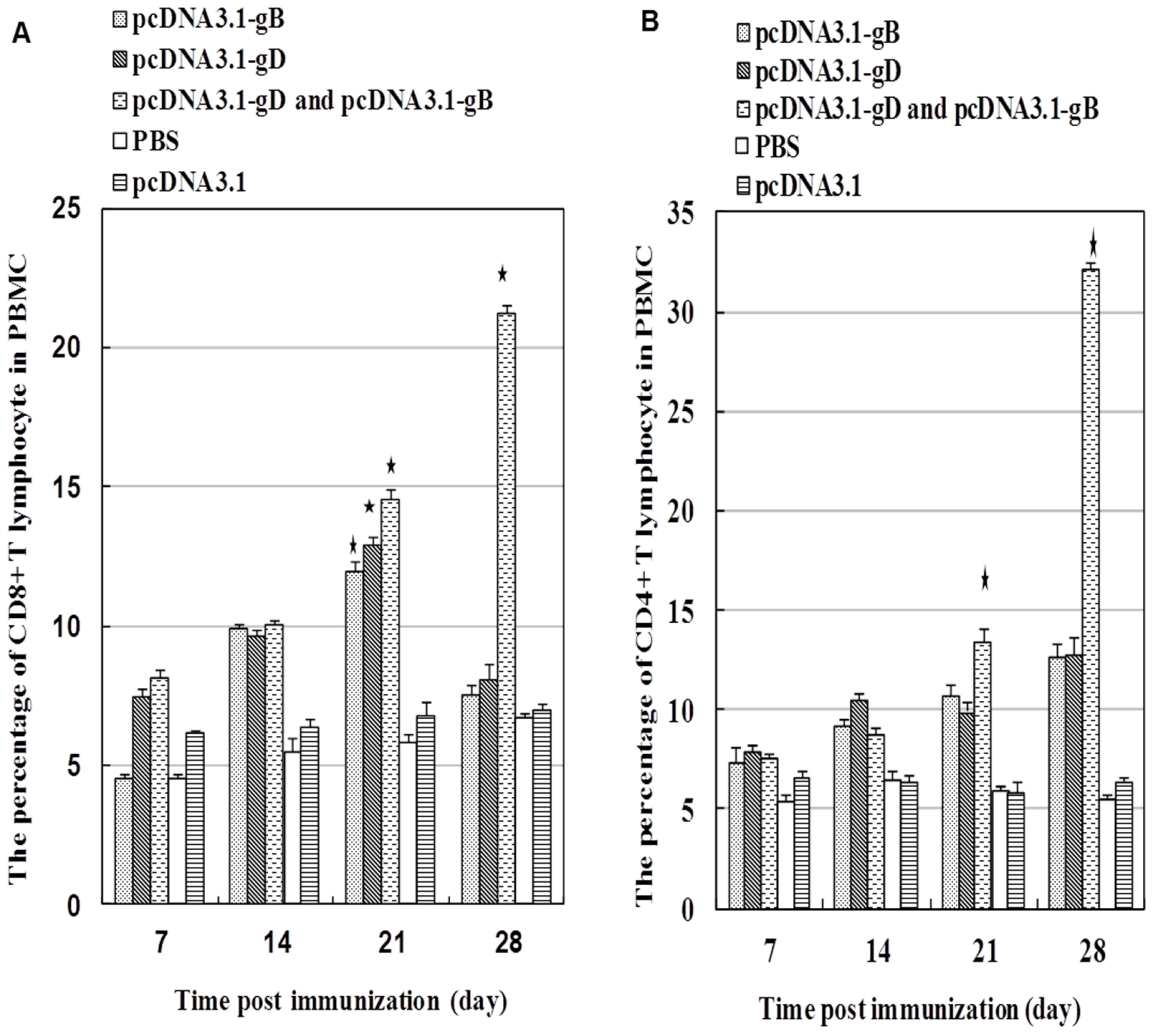
The frequency of CD4^+^ and CD8^+^ T cells in peripheral blood. Peripheral blood samples were obtained at the indicated time points post immunization, and the frequency of peripheral blood CD4^+^ and CD8^+^ T cells in different groups of Pekin ducks were characterized by flow cytometry analysis. Data are expressed as mean ± SD of the percentage of T cells in each group of Pekin ducks (n = 10 per group) from three separate experiments. A: The percentage of peripheral blood CD8^+^ T cells. B: The percentage of peripheral blood CD4^+^ T cells. * p<0.01 vs. the controls or unmarked groups at the same time point.

To test the efficacy of plasmid DNA vaccines, Pekin ducks were randomized and injected with control PBS or vaccinated with control plasmid of pcDNA3.1 or with monovalent or bivalent vaccine of gB and gD, respectively. The Pekin ducks were boosted with the same vaccine on day 14 post primary vaccination. Analysis of the frequency of peripheral blood CD4^+^ and CD8^+^ T lymphocytes revealed that the frequency of CD8^+^ T lymphocytes in the Pekin ducks vaccinated with monovalent or bivalent vaccine of gB and gD was significantly higher than those in the controls on day 21 post primary immunization, and the percentage of CD8^+^ T lymphocytes in the Pekin ducks vaccinated with bivalent vaccines remained significantly higher on day 28 post primary immunization (Figure 2). Furthermore, the frequency of CD4^+^ T lymphocytes in the Pekin ducks vaccinated with bivalent vaccines was significantly higher than those in the other groups of Pekin ducks on day 21 and 28 post primary immunization. The increased levels of CD4^+^ and CD8^+^ T cells suggested that vaccination with bivalent vaccines induced T cell responses in Pekin ducks.

### Vaccination with plasmids for gB and gD induces humoral responses in Pekin ducks

**Figure 3:**
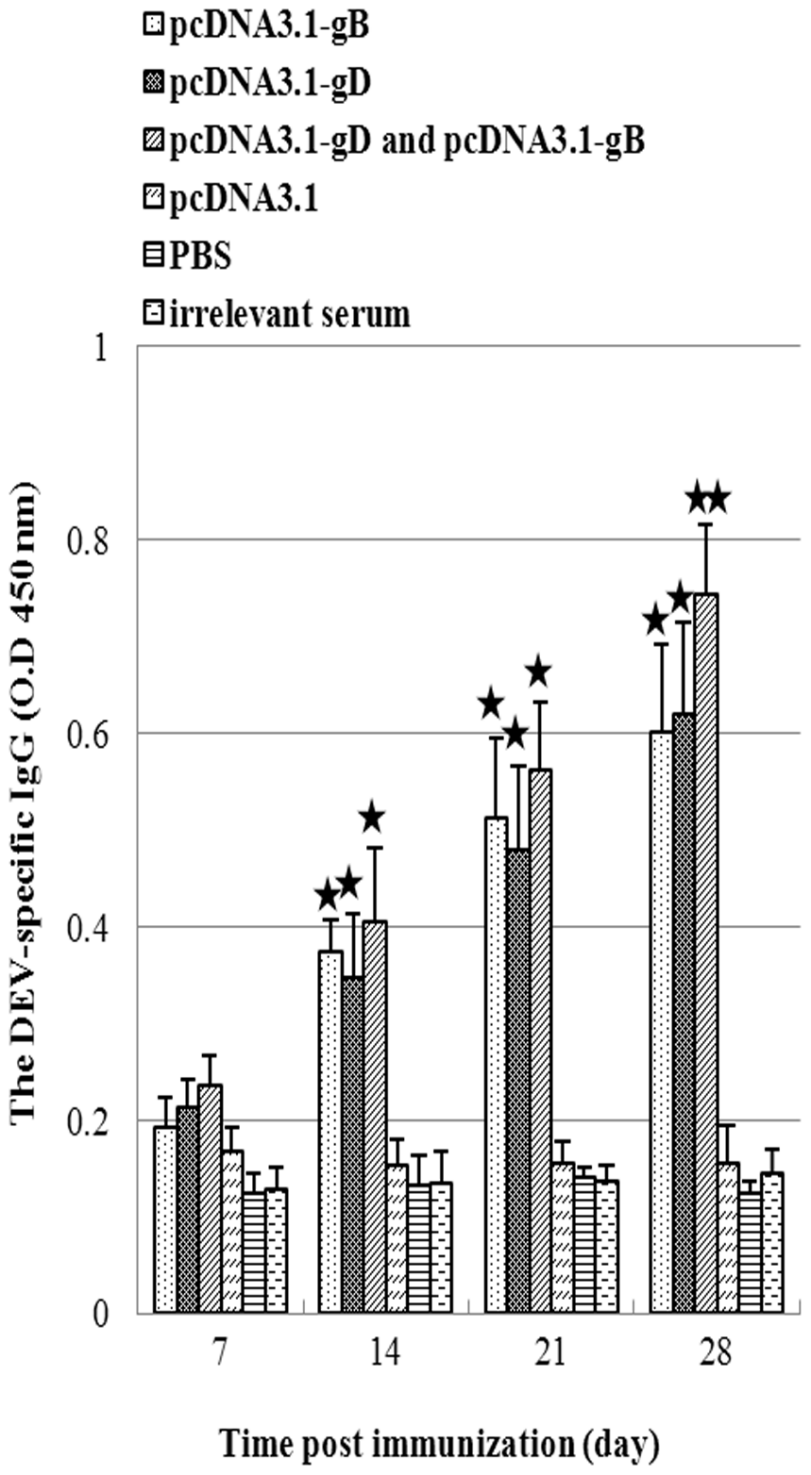
The levels of DEV-specific IgG responses. Blood samples were obtained from individual Pekin ducks at the indicated time points post immunization, and the levels of serum IgG antibodies against DEV were determined by ELISA. Data are expressed as mean ± SD of the levels of IgG in each group of Pekin ducks (n = 10, per group) from three separate experiments. * p<0.01 vs. the controls, ** p<0.05 vs. the groups marked *.

Next, we characterized humoral responses in Pekin ducks. While there was no detectable serum antibody against DEV from the PBS group, pcDNA3.1 group, and DHV group of animals, the levels of DEV-specific serum IgG response raised steadily in the three immunized groups (Figure 3) and were significantly higher than that in the PBS, pcDNA3.1, and DHV groups of animals when tested on day 14, 21 and 28 post vaccination (P<0.01). The mean titers of DEV-specific serum IgG antibodies were 1∶2048–3072 in three groups of animals at 21 days post vaccination and 1∶3072–4096 at 28 days post vaccination. There was no significant difference in the titers of IgG antibodies between the gB (1∶3072) and gD-vaccinated (1∶3276) animals. However, the titers of IgG antibodies were significantly higher in the bivalent vaccine group (1∶4096) than the monovalent groups of animals (p<0.05) on day 28. Analysis of the titers of DEV-specific neutralization antibodies indicated that the titers of DEV-specific neutralization antibodies in the Pekin ducks vaccinated with monovalent vaccine of gB or gD were at 1∶8 to 1∶16, while the titers of neutralization antibodies in the Pekin ducks vaccinated with bivalent vaccines reached 1∶32 to 1∶64 (Table 1). The titers of serum DEV-specific neutralization antibodies from the ducks receiving bivalent vaccines were significantly higher than that of those receiving monovalent vaccine (P<0.05). However, there was no significant difference in the titers of serum neutralization antibodies between two groups of ducks receiving different types of monovalent vaccine. These data indicated that vaccination with plasmids of pcDNA3.1-gB or pcDNA3.1-gD induced humoral responses, and vaccination with both plasmids enhanced humoral responses in Pekin ducks.

**Table 1:** The titers of neutralization antibodies before challenge.

group	PBS	pcDNA3.1	pcDNA3.1-gB	pcDNA3.1-gD	pcDNA3.1-gD and pcDNA3.1-gB^a^
neutralizing antibody titer	0	0	1∶8	1∶16	1∶32
	0	0	1∶8	1∶16	1∶32
	0	0	1∶16	1∶16	1∶32
	0	0	1∶16	1∶16	1∶32
	0	0	1∶16	1∶16	1∶64

Data are expressed as mean titers of two combined sera in the same group from three separate experiments.

a p<0.05 vs. the sera from the ducks receiving monovalent vaccine.

### Vaccination with plasmids for the DEV gB and gD protects from DEV challenge in Pekin ducks

**Figure 4:**
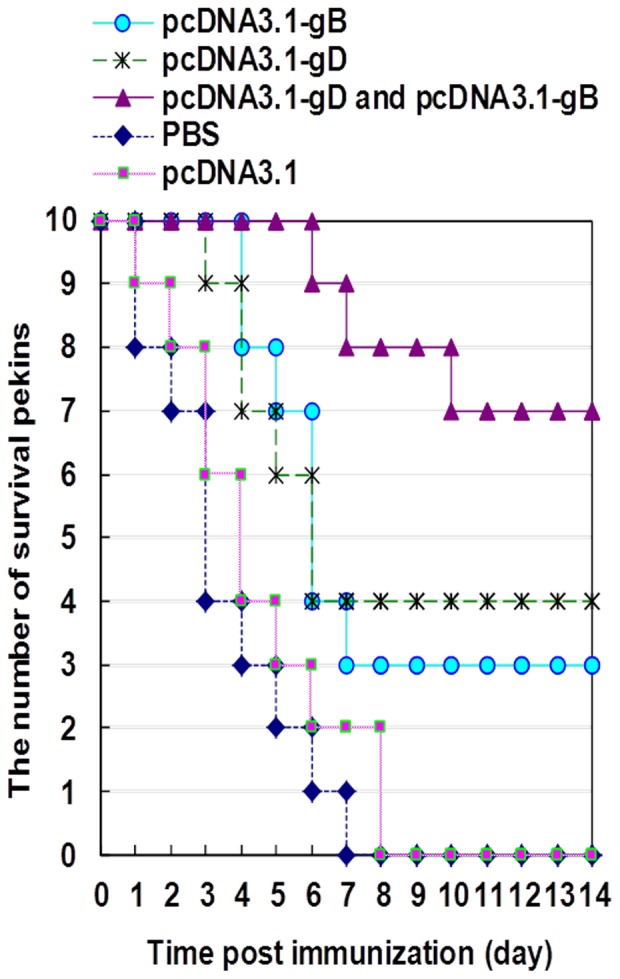
Vaccination with plasmids for the gB and gD protects against DEV infection in Pekin ducks. Groups of Pekin ducks were injected with PBS or control plasmid of pcDNA3.1 or vaccinated with monovalent or bivalent vaccines of gB and gD, respectively. The Pekin ducks were boosted with the same vaccine on day 14 post primary vaccination. Two weeks after the second vaccination, individual Pekin ducks were challenged with virulent DEV, and the survival of the different groups of Pekin ducks was recorded. Data shown are the mean time for the survival of each group of Pekin ducks (n = 10, per group).

To determine the efficacy of vaccination with the plasmids for the DEV gB and gD, groups of Pekin ducks were vaccinated with monovalent or bivalent vaccines of gB and gD, or injected with PBS or pcDNA3.1 as controls. The Pekin ducks were boosted with the same vaccine on day 14 post primary vaccination. Two weeks after the second vaccination, individual Pekin ducks were challenged with virulent DEV and monitored for the appearance of clinical symptoms, including inappetence, extreme thirst, droopiness, ataxia, ruffled feathers, nasal discharge, and watery diarrhea. We found that infection with DEV induced severe enteritis, which led to duck death. Control Pekin ducks started to die on day 1 post challenge and all of the Pekin ducks died during the observation period. In contrast, the Pekin ducks vaccinated with monovalent vaccine began to die on days 3–6 post challenge and 30–40% of the Pekin ducks survived throughout the observation period. More importantly, 70% of the Pekin ducks vaccinated with bivalent vaccines of gB and gD survived (Figure 4). The death rates in the different groups of ducks were significant difference on day 3 (p<0.05) and 8 (p<0.01) post challenge. Furthermore, the survival periods of the ducks receiving bivalent vaccines were significantly longer than that of those receiving monovalent vaccine (p<0.05). However, there was no significant difference in the survival period among the ducks receiving monovalent vaccine and controls (p>0.05). These data clearly indicated that vaccination with bivalent vaccines of gB and gD had better protection against DEV challenge in Pekin ducks.

## Discussion

The gB and gD of bovine herpes virus-1 have high immunogenicity and vaccination with the gB and gD can induce potent immunity against virulent virus infection in the field [26], [27], [28], [29], [30], [31]. Furthermore, vaccination with the gD of herpes simplex virus can reduce the recurrence and latency of herpes simplex virus in animal models [32], [33], [34]. In this study, we vaccinated the Pekin ducks with monovalent or bivalent vaccines of gB and gD for two times at a 2-week interval and tested the efficacy of vaccination with monovalent or bivalent vaccine of gB and gD in inducing immunity and protection against virulent DEV infection in Pekin ducks. We found that vaccination with monovalent vaccine of gB or gD induced potent immunity against DEV and that vaccination with bivalent vaccines of gB and gD stimulated stronger immune responses and had better protection against virulent DEV infection in Pekin ducks than monovalent vaccinations.

Th1 lymphocytes are major players in antiviral T cell immunity. The frequency of peripheral blood CD4^+^ and CD8^+^ T cells in the Pekin ducks vaccinated with a monovalent vaccine, particularly with bivalent vaccines, was also significantly higher than those in the controls after the primary and second vaccinations. Similarly, vaccination with bivalent vaccines induced much stronger peripheral blood T cell proliferation, in response to DEV (data not shown). Notably, activated CD4^+^ and CD8^+^ T cells can directly target the virus-infected cells and indirectly help B cells in producing neutralization antibodies.

The antigen-specific humoral responses, particularly against gB and gD, are crucial for the prevention of viral spreading. The enhanced T cell frequency should promote higher levels of humoral responses. We found that although there was no significant difference in the levels of serum DEV-specific IgG among groups of Pekin ducks vaccinated with monovalent or bivalent vaccines of gB and gD during the observation period, the titers of antibodies against DEV in the vaccinated Pekin ducks were significantly higher than that of the control groups. The titers of neutralization antibodies in the Pekin ducks vaccinated with bivalent vaccines of gB and gD were significantly higher than that in the Pekin ducks vaccinated with either monovalent vaccines. These data demonstrated that vaccination with bivalent vaccines induced stronger humoral responses.

More importantly, while challenge with virulent DEV in the control Pekin ducks injected with PBS or control plasmid induced severe enteritis and rapid death, infection with the virulent DEV in the Pekin ducks vaccinated with bivalent vaccines of gB and gD delayed the onset of enteritis. Furthermore, while all of Pekin ducks in the control groups infected with the virulent DEV died, some Pekin ducks, particularly for those vaccinated with bivalent vaccines, survived throughout the observation period. According to the China Institute of Veterinary Drugs Control established duck live vaccine manufacturing and inspection procedures, the ducks were injected 1000 lethal doses (LD) of DEV virulent strain, and observed for 10–14 days. The morbidity and mortality rates of Pekin ducks in the control group were 100% and 60%, while the onset of clinical symptoms in the vaccinated Pekin ducks delayed for a few days. In this study, control Pekin ducks died in 7 days, and the mortality rate was 100%, which was higher than that of 60% of previous observation. It suggests that the challenge dosage of DEV in this experiment may be higher than the standard one. Under this condition, 70% of the Pekin ducks vaccinated with bivalent vaccines of gB and gD survived, and apparently, vaccination with bivalent vaccines had stronger protection against DEV infection in Pekin ducks.

The potent protection is unlikely to be mediated by only higher levels of neutralization antibodies, because a previous study has shown that vaccination with gB and gD antigens induces strong humoral responses, but fails to protect against genital Herpes simplex virus (HSV) infection in humans [8]. Actually, antigen-specific T cell immunity is crucial for protection against HSV infection [34],[35]. It is possible that vaccination with bivalent vaccines induces potent T cell immunity and strong humoral responses, which contribute to the protection against DEV infection. Therefore, vaccination with DNA vaccines for multiple antigens may be a promising strategy for inducing potent immunity against DEV infection in birds. Previous studies have shown that intramuscular injection with 200 µg plasmid induces the highest titers of antibodies, and stronger cellular immune responses while vaccination with live attenuated vaccine predominantly induced humoral immune responses [36]. In addition, intramuscularly repeated vaccination with DNA vaccines at a 2-week interval induced potent cellular and humoral responses in animals [37], [38]. We used the same protocol to achieve a similar level of T cell and humoral responses to DEV in Pekin ducks.

## Conclusion

Our novel data demonstrated that vaccination with bivalent vaccines of gB and gD induced potent T cell immunity and strong humoral responses, and protected against the virulent DEV infection in Pekin ducks. Our findings may aid in the design of new vaccines for the prevention of DEV infection in animals.
